# EBF1, MYO6 and CALR expression levels predict therapeutic response in diffuse large B-cell lymphomas

**DOI:** 10.3389/fimmu.2023.1266265

**Published:** 2023-11-14

**Authors:** Alice Turdo, Miriam Gaggianesi, Caterina D’Accardo, Gaetana Porcelli, Sebastiano Di Bella, Dario Cricchio, Irene Pillitteri, Rossana Porcasi, Melania Lo Iacono, Francesco Verona, Chiara Modica, Narges Roozafzay, Ada Maria Florena, Giorgio Stassi, Salvatrice Mancuso, Matilde Todaro

**Affiliations:** ^1^ Department of Health Promotion, Mother and Child Care, Internal Medicine and Medical Specialties (PROMISE), University of Palermo, Palermo, Italy; ^2^ Department of Surgical, Oncological and Stomatological Sciences (DICHIRONS), University of Palermo, Palermo, Italy; ^3^ A.O.U.P. “Paolo Giaccone”, University of Palermo, Palermo, Italy

**Keywords:** diffuse large B-cell lymphoma, R-CHOP, therapy resistance, elderly patients, gene expression signature, biomarkers of response

## Abstract

**Background:**

Diffuse large B-cell lymphoma (DLBCL) is a hematological malignancy representing one-third of non-Hodgkin’s lymphoma cases. Notwithstanding immunotherapy in combination with chemotherapy (R-CHOP) is an effective therapeutic approach for DLBCL, a subset of patients encounters treatment resistance, leading to low survival rates. Thus, there is an urgent need to identify predictive biomarkers for DLBCL including the elderly population, which represents the fastest-growing segment of the population in Western countries.

**Methods:**

Gene expression profiles of *n*=414 DLBCL biopsies were retrieved from the public dataset GSE10846. Differentially expressed genes (DEGs) (fold change >1.4, p-value <0.05, n=387) have been clustered in responder and non-responder patient cohorts. An enrichment analysis has been performed on the top 30 up-regulated genes of responder and non-responder patients to identify the signatures involved in gene ontology (MSigDB). The more significantly up-regulated DEGs have been validated in our independent collection of formalin-fixed paraffin-embedded (FFPE) biopsy samples of elderly DLBCL patients, treated with R-CHOP as first-line therapy.

**Results:**

From the analysis of two independent cohorts of DLBCL patients emerged a gene signature able to predict the response to R-CHOP therapy. In detail, expression levels of EBF1, MYO6, CALR are associated with a significant worse overall survival.

**Conclusions:**

These results pave the way for a novel characterization of DLBCL biomarkers, aiding the stratification of responder *versus* non-responder patients.

## 1 Introduction

Diffuse large B-cell lymphoma (DLBCL) is the most common subtype of non-Hodgkin’s lymphoma and one of the highest mortality rates for all countries in the world within the elderly subjects (1). DLBCL is a heterogeneous disease at molecular and genetic level, characterized by a different biological behavior.

Although more than 50% of patients affected by DLBCL successfully respond to standard therapy, approximately 40% experience a relapse, making this neoplasia the leading cause of morbidity due to limited treatment options (2). Moreover, DLBCL commonly occurs in patients with comorbidities or in very elderly patients who warrants geriatric assessment prior treatment. Thus, a comprehensive examination of treatment efficacy versus the occurrence of side effects is required in order to predict tolerability, cardiotoxicity and the broad quality of life in frail patients (3).

Several studies have shown that the magnitude of clinical benefit rate in therapies for the treatment of DLBCL, which is mainly based on the use of immunotherapy in combination with chemotherapy (R-CHOP), reflects the molecular heterogeneity, including gene copy-number alterations and mutations (2, 4). Of note, in the last decades, the addition of rituximab to the standard CHOP therapy, significantly improved, by 10-15%, the overall survival of DLBCL patients (2).

Nonetheless comprehensive mechanisms underlying the refractoriness to R-CHOP have not been determined, several clinical parameters have been associated with treatment resistance and worse outcomes. The main prognostic model applied to DLBCL is based on the International Prognostic Index (IPI). The scoring system allows to stratify patients from low risk (0/1 score) to high risk (4/5 score) groups, depending on age, serum lactate dehydrogenase (LDH) levels, the eastern cooperative oncology group (ECOG) performance status, number of extranodal sites and Ann Arbor stage disease (5). Newly diagnosed DLBCL patients, treated with R-CHOP, are categorized according to the revised IPI, which facilitates the prognostic classification of patients (6).

Apart from the scoring system incorporating clinical parameters, advances in molecular characterization led to distinguish two different molecular subtypes of DLBCLs with a different biological behavior, the germinal center B-cell (GCB) lymphoma and the activated B-cell (ABC) lymphoma, this last associated with a poorer prognosis. These molecular subtypes of DLBCL are likewise arising from distinct cell of origin at diverse stages of lymphoid differentiation and specifically GCB from normal germinal-center B cells, while ABC from a post-germinal B cell (7).

Although several integrative approaches and models to detect patients at increased risk of relapse have been proposed, the identification of decisive driver biomarkers that can predict therapy response is still an unmet need. In the present study, in order to identify the gene expression profile of elderly (≥65-year-old) DLBCL patient’s responders and non-responders to the therapy with CHOP and R-CHOP, we benefited from a publicly available dataset (GSE10846) (7). Using a multiplexed gene expression analysis, furtherly validated by immunohistochemical evaluation, it has been identified a gene signature predictive of therapeutic response, in an independent cohort of DLBCL patients. From the molecular analysis emerged that expression of EBF1, MYO6 and CALR is able to select patients with distinct outcomes. Here, we provided biomarkers that could be of clinical interest to stratify elderly DLBCL patients, predicting the response to standard therapy, and develop novel therapeutic strategies based on the knowledge acquired, regarding validated molecular targets.

## 2 Materials and methods

### 2.1 Study populations

DLBCL tumor specimens and patients’ clinical data were obtained at the Hematology Unit, “P. Giaccone” Hospital of Palermo. Elderly patients (≥ 65-year-old) have been selected for the study and further classified in two cohorts of responder (n=13) and non-responder (n=6) to first-line R-CHOP therapy (validation cohort). A panel of hematologist and pathologist at the “P. Giaccone” Hospital followed the ESMO Clinical Practice Guidelines and Italian Society of Hematology guidelines for diagnosis, treatment and follow-up of DLBCL patients.

### 2.2 Statistical analysis

The training cohort (GSE10846, *n*=414) has been divided in two groups: patients treated with CHOP (*n*=181) and patients treated with R-CHOP (*n*=233). Patients’ cohort has been filtered by age (≥ 65-year-old) (*n*=188) and subsequently divided in responder (*n*=94) and non-responder (*n*=94) (7).Differential expressed genes (DEGs) (fold change >1.4, p-value <0.05, *κ*=387) have been clustered in the responder and non-responder groups and according to LDH levels. The activated B-cell (ABC) and germinal center B-cell (GCB) molecular subtypes have been reported as annotations.

Finally, the top 30 up-regulated genes of responder and non-responder patients have been used to perform enrichment analysis in order to identify the main signatures involved in gene ontology (MSigDB), considering molecular function, biological process and cellular component (p-value<10^-7^). The signatures associated with the first 30 upregulated genes were also computed by the QIAGEN Ingenuity Pathway Analysis software.

The association between features and patients’ overall survival was assessed by using Cox proportional- hazards model. Specifically, in the univariate analysis Cell of origin (COO), ECOG performance status, Extranodal Sites, IPI, LDH, sex and stage parameters were dichotomized according to (8, 9). The dichotomization of the identified signature was defined by using the median expression of each gene (*MYO6*, *EBF1* and *CALR*). In the multivariate analysis, we combined our signature with each previously described feature.

To generate the Kaplan-Meier curves of overall survival by using the GSE10846 dataset, the initial population was filtered by age (≥ 65-year-old). “High” and “Low” groups were defined by using the median expression of each gene (*MYO6*, *EBF1* and *CALR*) in the patient cohort.

All analyses were performed with R survival, survminer, and coxph libraries. Graphs were created by using the ggplot2 library.

### 2.3 RNA extraction and droplet digital PCR

Total RNA from FFPE tumor tissue specimens was isolated by using RNeasy FFPE Kit (Qiagen). 300 ng of total RNA was retro-transcribed with the high-capacity c-DNA reverse transcription kit (Applied Biosystem). In order to perform a four-gene multiplex assay, we used specific Droplet digital PCR (ddPCR- QX200 Droplet Reader) gene expression assays with FAM (*n=2*) and HEX (*n=2*) fluorophores. To optimize the multiplex reactions, from 100 to 300nM gene-specific primers have been used in combination with ddPCR supermix for probes (No-dUTP) and 25 ng of cDNA samples. Droplets were generated using the QX200 Droplet Generator (Bio-Rad) and dispensed into a 96 well-PCR plate. PCRs were performed in a ProFlex PCR System (Applied Biosystem) with the following protocol: 1x (95°C for 10 min), 50x (94°C for 30 sec, 56°C for 1 min), 1x (98°C for 10 min). After gene target amplification, samples were analyzed using QX200 Digital Droplet Reader (Bio-Rad). Gene expression analyses (copies/µl) were performed using QX Manager Software (1.2 Standard Edition) and normalized by using *GAPDH*.

### 2.4 Immunohistochemistry

FFPE lymphoma tissue specimens, stratified by age ≥ 65-year-old, were obtained from 11 responder patients and 4 non responder patients treated with R-CHOP.

Antigen retrieval was performed using the PT link system (Dako, Agilent Technologies, Santa Clara, CA, USA). Thereafter, sections were permeabilized with the 0.1% TRITON X-100 PBS for 10 min on ice, followed by 3% H_2_0_2_ and 10% human serum blocking incubation.

All slides were exposed overnight at 4°C to primary antibodies against Calreticulin (CAL-R) (ab22683; mouse IgG1; Abcam, Cambridge Science Park, UK), Myosin VI (MYO6) (MUD-19; mouse IgG1, Sigma-Aldrich), and EBF-1 (HPA061169; rabbit; Sigma-Aldrich). Staining was revealed using a biotin-streptavidin system (Dako LSAB2 System-HRP) and detected with the DAB substrate chromogen system (Dako). Nuclei were counterstained with Mayer’s Hematoxylin (Lillie’s Modification) Histological Staining Reagent (Dako). Staining was analyzed using an Olympus BX60 microscope. Immunohistochemical analysis were quantified with Image J.

## 3 Results

### 3.1 The analysis of a large cohort of elderly DLBCL patients revealed a gene signature associated to prognosis

**Figure 1:**
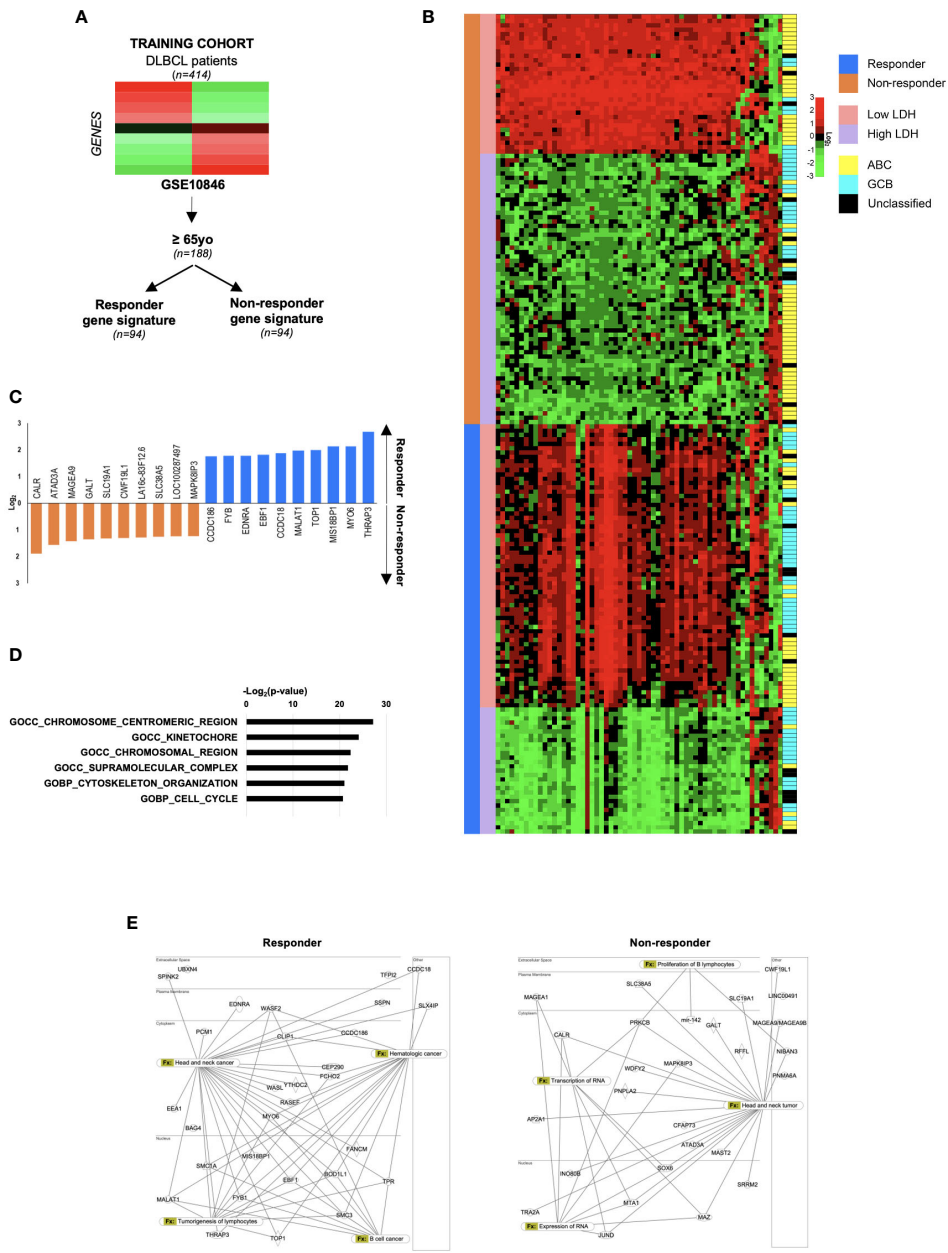
Gene expression analysis of a large cohort of DLBCL patients reveal a gene signature associated to prognosis. **(A)** Workflow chart indicating the process to select DLBCL responder and non-responder gene signatures in the training cohort of the GSE10846 database. **(B)** Heatmap of differential expressed genes (DEGs) (fold change >1.4, p-value <0.05, *κ*=387) in responder *versus* non responders DLBCL patients. The LDH levels and the molecular subtype classification are shown. **(C)** Top ten up-regulated genes (log2) in responder (blue) and non-responder (orange) DLBCL patient cohort. **(D)** Enrichment analysis in gene ontology (MSigDB) in responder and non-responder DLBCL patient cohort. **(E)** Protein network analysis, generated with DEGs listed in **Supplementary Table 1** The networks were generated through the use of QIAGEN IPA (QIAGEN Inc., https://digitalinsights.qiagen.com/IPA), in responder and non-responder DLBCL patient cohort.

Diffuse large B-cell lymphoma (DLBCL) is a heterogeneous disease, causing high mortality in elderly patients. Despite the adverse effects, CHOP- and R-CHOP-based therapies result effective in the two-thirds of DLBCL patients, of which the rest portion experiences disease recurrence. Being DLBCL elderly patients the more susceptible to standard therapy side effects, in order to identify the genes predictive of therapy response, a gene expression analysis of 188 pretreatment biopsies of patients with an age ≥ 65 was retrieved from a publicly available dataset (GSE10846) (**Figure 1A**) (7).

Following unsupervised hierarchical clustering, the dichotomization of training cohort patients in responder and non-responder to CHOP and R-CHOP therapy allowed the identification of differentially expressed genes (DEGs) associated to a poor outcome (**Figure 1B**). The median age of the responder and non-responder cohorts of patients, to standard therapy, was comparable (74.04 *versus* 75.12-year-old) thus allowing the exclusion of age-related deaths. Of note, responder patients were mainly characterized by the GCB-like (62,5%) molecular subtype associated with a favorable outcome (**Figure 1B**). In accordance with well-established negative prognostic LDH parameter, responder DLBCL patients harbored lower LDH levels (1,0887 *versus* 1,8112) with respect to non-responder patients (**Figure 1B**; **Supplementary Figures S1A, B**). Analysis of gene expression profile, including 387 genes, of responder *versus* non-responder patients showed ten most differentially expressed genes (p-value ≤ 0.001) (**Figure 1C**; **Supplementary Table 1**). Specifically, *ATAD3A, CALR*, *CWF19L1, GALT, MAGEA9, MAPKI8IP3, PSLNR, SEPTIN7P13, SLC19A1* and *SLC38A5* resulted up-regulated in non-responder patients, while high expression levels of *EBF1*, *EDNRA*, *CCDC18*, *CCDC186, FYB*, *MALAT1*, *MIS18BP1*, *MYO6*, *THRAP3* and *TOP1*, characterized responder DLBCL patients (**Figure 1C**).

The enrichment analysis of top 30 upregulated genes computed with Molecular Signatures Database (MSigDB), revealed six signatures associated with cell cycle, cell division and cytoskeleton organization, which are related to B cell malignant neoplasia (**Figures 1D, E**; **Supplementary Table 2**). Together these data provide evidence that these gene signatures may select responder from non-responder patients, identifying patients with a better life expectancy.

### 3.2 The expression levels of three genes predicted the response of DLBCL patients to R-CHOP therapy

In order to validate the expression levels of previously identified genes in dictating the dichotomization in life expectancy, we analyzed a cohort of naïve DLBCL patients, diagnosed and in follow-up at the Hematology/Oncology Unit of the “P. Giaccone” Hospital in Palermo, treated with R-CHOP as first -line therapy (validation cohort), selecting the frail cohort of DLBCL patients (≥ 65-year-old) (**Table 1**).

**Table 1:** Clinical parameters of patients with Diffuse Large B Cell Lymphoma (DLBCL) treated with rituximab, cyclophosphamide, doxorubicin, vincristine, and prednisone (R-CHOP) therapy.

Patient #	Age	Sex	Ann Arbor Stage	ECOG	Extranodal site	LDH	IPI	Responder
1	68	F	I	1	0	269	2	Yes
2	65	M	II	1	0	437	1	Yes
3	68	F	I	1	0	174	1	Yes
4	67	M	III	1	0	452	3	Yes
5	67	F	III	1	1	374	3	Yes
6	73	M	I	1	1	142	1	Yes
7	72	F	nd	nd	0	nd	nd	Yes
8	76	M	I	1	1	149	1	Yes
9	69	F	III	1	0	274	2	Yes
10	67	F	IV	1	0	985	3	Yes
11	69	F	IV	1	0	651	nd	Yes
12	79	M	IV	1	0	260	3	No
13	70	M	IV	2	1	511	5	No
14	71	M	IV	1	1	372	3	No
15	73	F	II	nd	nd	nd	1	No
16	73	F	nd	1	0	nd	1	Yes
17	69	M	nd	1	0	nd	3	Yes
18	65	M	nd	3	0	nd	4	No
19	70	F	nd	3	0	nd	2	No

Age is referred to the time of diagnosis. International prognostic Index (IPI) score varies from 0 to 5, according to the presence of prognostic factors. Responder patients to R-CHOP therapy are indicated with “Yes”, while non-responder patients to R-CHOP therapy are indicated with “No”.

Nd, not determined.

**Figure 2:**
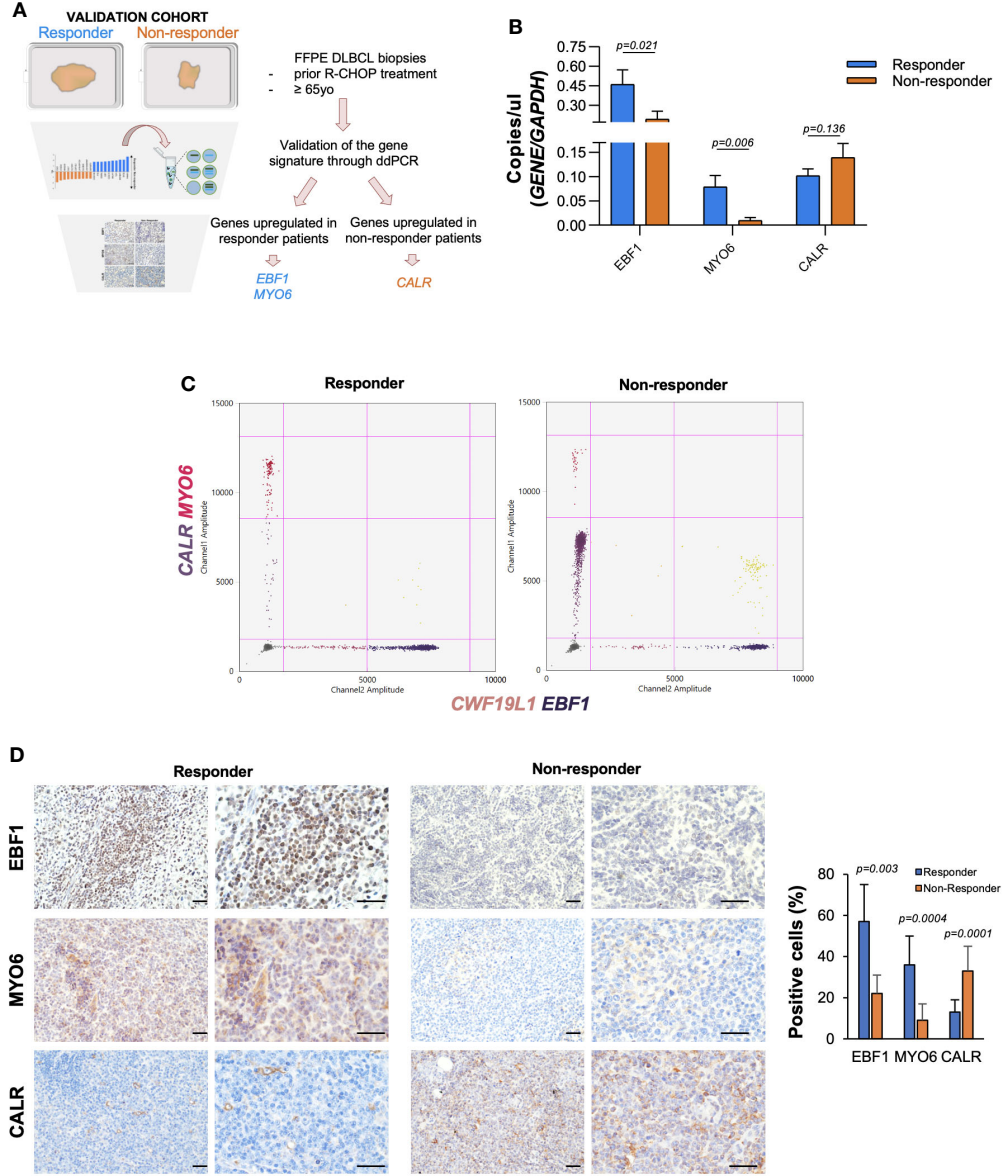
EBF1 and MYO6 result highly expressed in responder DLBCL patients whilst CALR high expression characterize non responder DLBCL patients. **(A)** Workflow chart indicating the validation of the identified signature in our cohort of FFPE DLBCL samples. **(B)** Absolute mRNA levels (copies/µl) of EBF1, MYO6 and CALR in responder and non-responder DLBCL patients (*n*=19). Data are represented as mean  ±  SEM of three independent experiments. **(C)** Representative droplet digital PCR (ddPCR) scatter plots showing single, double, triple or quadruple positive droplets for EBF1, MYO6, CALR and CWF19L1 of FFPE samples of responder (pt#1) and non-responder DLBCL patient (pt#14). **(D)** Representative IHC analysis for EBF1, MYO6 and CALR of patients as in **(C)** Scale bar is 100 µm.

To overcome the low abundance and integrity of RNA content on formalin-fixed paraffin embedded (FFPE) biopsy samples, we adopted an implemented quantitative multiplex droplet digital PCR-based assay (**Figure 1C**; **Figure 2A**), from which emerged that two out ten DEGs, previously identified, *EBF1* and *MYO6* resulted up-regulated, at both mRNA and protein levels, in responder DLBCL patients (**Figures 2B–D**; **Supplementary Figures 2A, B**; **Supplementary Table 3**). Furthermore, the gene expression analysis of the ten upregulated genes, arisen from the non-responder included in the dataset (GSE10846), displayed an increasing trend of CALR mRNA levels, although not reaching statistical significance, which was paralleled by high protein expression levels (**Figures 2B–D**; **Supplementary Table 4**). These data indicate that EBF1, MYO6 and CALR could predict DLBCL patients’ response to R-CHOP therapy and aid in the stratification of responder *versus* non-responder patients.

### 3.3 EBF1, MYO6 and CALR signature is associated with survival probability in DLBCL patients

To investigate the clinical significance of the identified signature, the magnitude of *EBF1*, *MYO6*, and *CARL* expression levels has been correlated to the survival data of ≥ 65-year-old DLBCL patients of the training cohort.

**Figure 3:**
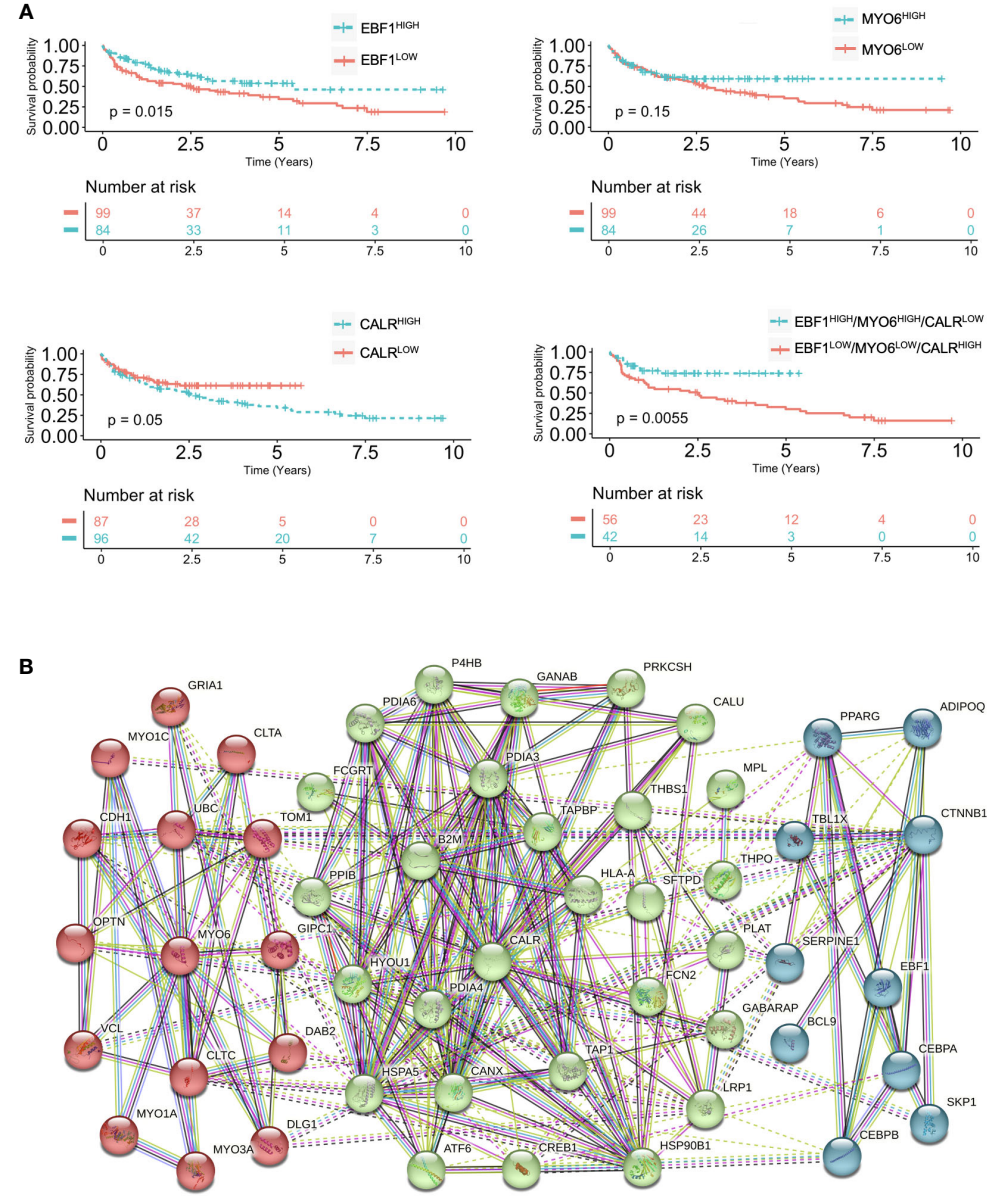
Expression of EBF1, MYO6 and CALR correlate with prognosis in DLBCL. **(A)** Kaplan Meier overall survival (OS) curves of elderly DLBCL patients (GSE10846) stratified by high or low EBF1, MYO6 and CALR expression levels. **(B)** Functional protein association network of EBF1, MYO6 and CALR based on STRING database.

Transcriptome microarray analysis of a cohort of 154 DLBCL revealed a significant negative correlation between the signature EBF1^low^, MYO6^low^ and CALR^high^ expression and survival probability of patients, assuming a more pronounced significance than single gene expression (**Figure 3A**). Univariate analysis denoted that EBF1^high^, MYO6^high^ and CALR^low^ signature expression is an independent positive prognostic factor of overall survival showing a higher statistical significance over several important clinical parameters, such as ECOG performance status, extranodal sites and stage (**Supplementary Figure 3A**). Importantly, EBF1^high^, MYO6^high^ and CALR^low^ signature expression significantly increased the prognostic value of ECOG performance status, extranodal sites, IPI, LDH and stage (**Supplementary Figure 3B**).

In line with the signature prognostic value, from the STRING network analysis emerged an implication of EBF1, MYO6, and CALR in the regulation of tumor progression together with the control of B lymphocyte gene transcription, intracellular vesicle transport and protein folding (**Figure 3B**; **Supplementary Figure S3C**). Being restricted the signature expression to DLBCLs as compared to non-tumoral lymphoid cells, a specific targeting of EBF1, MYO6 and CALR could be exploited for therapeutic intervention (**Supplementary Figure S4A**).

**Figure 4:**
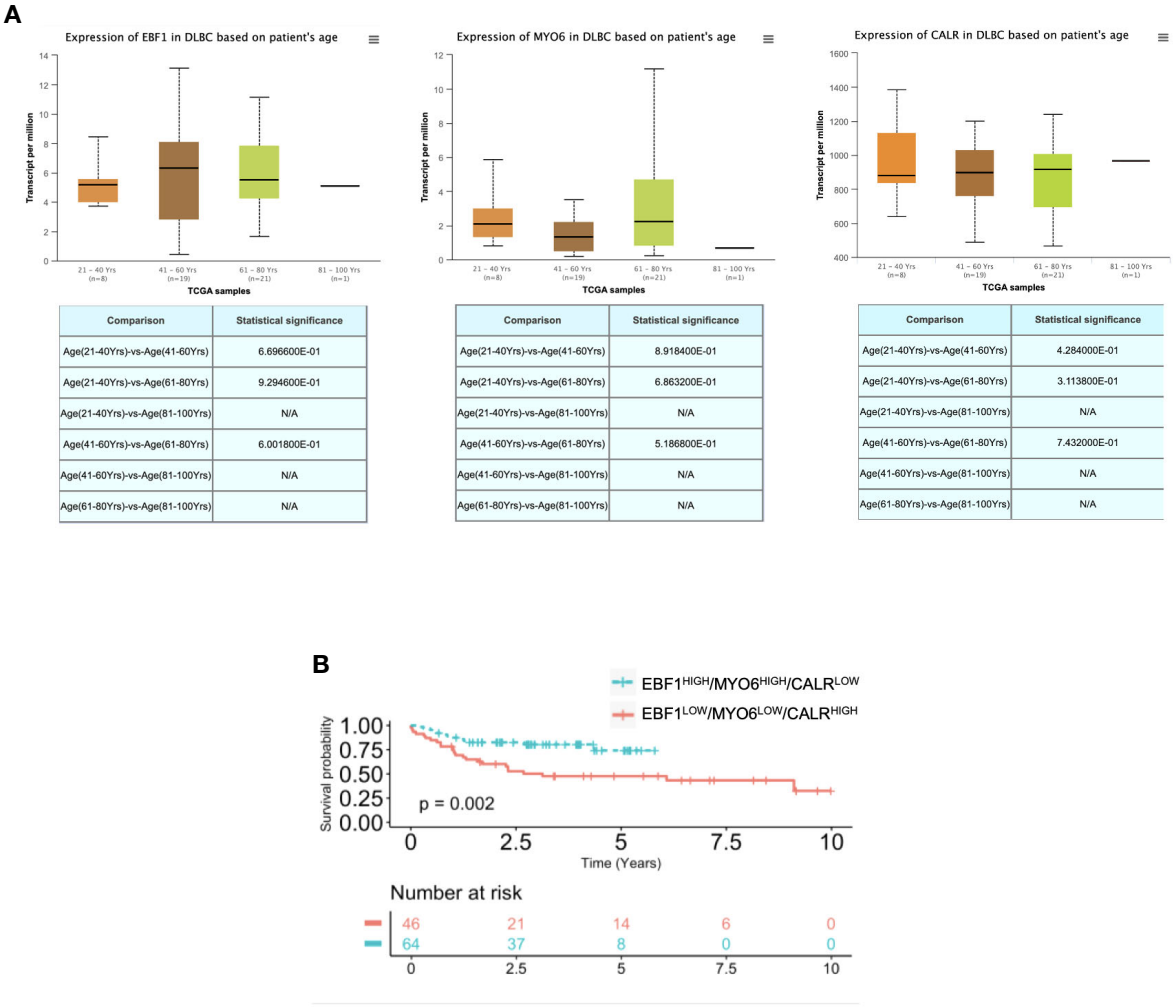
EBF1, MYO6 and CALR characterize DLBCL as compared to normal lymphoid tissues. **(A)** RNA seq expression data of EBF1, MYO6 and CALR in DLBCL patients at different age, retrieved from the TCGA database and analyzed by UALCAN. **(B)** Kaplan Meier overall survival (OS) curves of adult DLBCL patients (<65-year-old) (GSE10846) stratified by high or low EBF1, MYO6 and CALR expression levels. N/A stands for not available.

To investigate whether the *EBF1*, *MYO6* and *CALR* differential expression could be an age-independent signature to predict R-CHOP response, we assessed their expression levels in a heterogeneous age group, which revealed a comparable expression level in adults as well as elderly DLBCL patients (**Figure 4A**). Of note, beside in the over 65-year-old, EBF1^low^, MYO6^low^ and CALR^high^ signature expression is able to dichotomize the response to R-CHOP treatment in under 65-year-old DLBCL patients (**Figure 4B**; **Supplementary Figure S4B**). Although EBF1^high^, MYO6^high^ and CALR^low^ signature expression was an independent positive prognostic factor in under 65-year-old DLBCL patients, it did not increase the prognostic value of the selected clinical features in a multivariate analysis (**Supplementary Figures 4C, D**).

Our study unveils a robust predictive and prognostic signature able to determine the response to R-CHOP treatment in both under and over 65-year-old DLBCL patients. Notably, EBF1^high^, MYO6^high^ and CALR^low^ signature expression ameliorates the prognostic power of the most important clinical parameters and, in particular, of the IPI clinical risk scoring system in the elderly patients.

## 4 Discussion

The clinical use of the so far identified gene signatures, mostly associated with tumor microenvironmental components, led to unsatisfied clinical outcomes of several DLBCL patients that remains poor (7, 10). Thus, it is becoming increasingly clear that the heterogeneity of patients, affected by DLBCL, has been underestimated, posing an urgent need to identify novel specific biomarkers for predicting the response to standard therapy.

Here, we found a new signature that significantly associates with the progression of disease that may be exploited for curative therapies in advanced DLBCLs. Droplet digital PCR and immunohistochemistry analysis of a cohort of ≥65-year-old naïve DLBCL patients revealed that EBF1, MYO6 and CALR expression levels stratify patients for the response to the standard R-CHOP therapy, regardless the IPI score, currently used in clinical settings. MYO6 is a motor protein, classified as unconventional myosin protein due to its reverse direction movement towards the actin filaments. MYO6 is involved in vesicular and macromolecules transport, cell migration and signaling (11). Albeit being implicated in prostate and breast cancer progression, here we uncover a novel role as favorable predictive biomarker in DLBCLs. CALR controls the protein folding by regulating the protein glucosylation-deglucosylation cycle and calcium homeostasis in the endoplasmic reticulum (12). CALR genetic alterations have been observed in several cancer types and correlated to a worse outcome. Specifically, CALR driver mutations has been described in myeloproliferative disorders but have not yet been associated to other hematological neoplasia (13, 14). Interestingly, the main driver mutations in CALR exon 9 change protein localization, from a cytoplasmic form to a membrane bound homodimers (15). Secretion of mutated CALR has also been observed in liquid biopsies samples as urine in bladder urothelial cancer patients (16). Our pioneer findings prospectively pose CALR as an extremely powerful biomarker in DLBCL patients and further analysis are necessary to clarify the mutational status of CALR also in DLBCL patients.

Being EBF1, together with E2A and Pax5, involved in the B-cell lineage commitment by regulating cell transcription, it has been also implicated in the development of B-cell-acute lymphoblastic leukemias (B-ALL) (17). EBF1 protein levels have been otherwise associated to better prognosis in colorectal cancer and cholangiocarcinoma and to a worse outcome in triple-negative breast cancer (18–20). The herein reported findings indicate that the *EBF1^low^/MYO^low^/CALR^high^* gene expression foresees the failure in therapeutic response of patients associated with a low-intermediate IPI risk, airing this identified signature as significant prognostic power. While further studies are needed to investigate MYO6 regulation of lymphoma cell dynamics and to design novel therapeutic approaches, a monoclonal antibody targeting the neoepitope generated by mutated CALR in myeloproliferative disorders has been generated. This approach could be prospectively applied to DLBCL patients harboring alterations in *CALR* (21). To date, specific agonists of EBF1 are not clinically available, however, evidence showed that the inhibition of EBF1 may be influenced by Notch and IL-7 signaling, whose modulation by already accessible compounds could indirectly interfere with EBF1 (17). Our findings provide evidence that EBF1, likely by preserving B cell entity, is required together with MYO and CALR for R-CHOP response and significantly associates with patient overall survival.

High-grade DLBCLs requires more intensive therapies mainly characterized by the addition of other chemotherapeutic treatments. Although the major limitation of this study relies in the lack of functional validation of the newly identified genes, EBF1, MYO6 and CALR could be considered targetable candidates to aid advanced DLBCL management.

## Data availability statement

The raw data supporting the conclusions of this article will be made available by the authors, without undue reservation.

## Ethics statement

Ethical approval was not required for the studies involving humans because the study was performed on already published dataset and retrospective samples. The studies were conducted in accordance with the local legislation and institutional requirements. For the human samples used in this study, a written informed consent was not required from the participants or the participants' legal guardians/next of kin in accordance with the national legislation and the institutional requirements. 

## Author contributions

AT: Conceptualization, Funding acquisition, Supervision, Writing – original draft, Writing – review & editing. MG: Conceptualization, Funding acquisition, Supervision, Writing – original draft, Writing – review & editing. CD’A: Methodology, Writing – review & editing. GP: Methodology, Writing – review & editing. SB: Methodology, Writing – review & editing. DC: Methodology, Writing – review & editing. IP: Resources, Writing – review & editing. RP: Resources, Writing – review & editing. MLI: Methodology, Writing – review & editing. FV: Methodology, Writing – review & editing. CM: Methodology, Writing – review & editing. NR: Methodology, Writing – review & editing. AF: Resources, Writing – review & editing. GS: Funding acquisition, Writing – review & editing, Writing – original draft. SM: Funding acquisition, Writing – review & editing, Writing – original draft. MT: Conceptualization, Funding acquisition, Writing – original draft, Writing – review & editing.
