# Supplementary material for: EBF1, MYO6 and CALR expression levels predict therapeutic response in diffuse large B-cell lymphomas
Source: Front Immunol. 2023 Nov 14;14:1266265. doi: 10.3389/fimmu.2023.1266265 (PMC10682075; doi:10.3389/fimmu.2023.1266265)
Supplement: Supplementary file 1 [file DataSheet_1.pdf]

Supplementary Material

EBF1, MYO6 and CALR expression levels predict survival in diffuse large B-cell lymphomas

Supplementary Figure S1. DLBCL patient poor prognosis correlated to high LDH levels.

(A, B) LDH high and low stratification in responder (A) and in non-responder (B) DLBCL patients, based on LDH mean level expression.

Supplementary Figure S2. EBF1, MYO6 and CALR expression in positive and negative controls.

(A) EBF1, MYO6 and CALR immunohistochemical analysis on lymph node, prostate cancer, and placenta, respectively. Isotype-specific immunoglobulins were used as negative control. Scale bar is 100 µm. **(B)** Table showing the number of responder and non-responder DLBCL patients characterized by $EBF1^{high}/MYO6^{high}/CALR^{low}$ and $EBF1^{low}/MYO6^{low}/CALR^{high}$.

Supplementary Figure S3. EBF1, MYO6 and CALR are involved in pathways regulating cell proliferation and migration and B cell differentiation.

(A) Univariate analysis of demographic and clinical parameters and the identified gene signature for overall survival in over 65-year-old DLBCL patients. **(B)** Multivariate analysis of the identified gene signature and each clinical parameter as in (A). HR = hazard ratio, CI = confidence interval, ECOG = Eastern Cooperative Oncology Group, $EBF1^{low}/MYO6^{low}/CALR^{high}$ = $EBF1^{L}/MYO^{L}/CALR^{H}$, $EBF1^{high}/MYO6^{high}/CALR^{low}$ = $EBF1^{H}/MYO^{H}/CALR^{L}$. **(C)** Functional protein association network of EBF1, MYO6 and CALR based on STRING database.

Supplementary Figure S4. EBF1, MYO6 and CALR expression characterize lymphoid neoplasia. **(A)** Box plot representing the distribution of log2 (TPM+1) gene expression of EBF1, MYO6 and CALR in lymphomas (T) (*n*=47) versus normal lymphoid tissues (N) (*n*=337) retrieved from (GEPIA) (1). p value (<0.01) was calculated with one-way ANOVA test. **(B)** Kaplan Meier overall survival (OS) curves of DLBCL patients at any age (GSE10846) stratified by high or low EBF1, MYO6 and CALR expression levels. **(C)** Univariate analysis of demographic and clinical parameters and the identified gene signature for overall survival in under 65-year-old DLBCL patients. **(D)** Multivariate analysis of the identified gene signature and each clinical parameter as in (C).

Responder		Non-responder	
GENE	$\log_2$ FC	GENE	$\log_2$ FC
THRAP3	2.68546	CALR	1.88754
MYO6	2.13115	ATAD3A	1.56093
MIS18BP1	2.12645	MAGEA9	1.42204
TOP1	1.98763	GALT	1.34994
MALAT1*	1.96538	SLC19A1*	1.31763
CCDC18	1.86677	CWF19L1	1.30102
EBF1	1.81487	LA16c-83F12.6*	1.28188
EDNRA	1.77893	SLC38A5	1.25407
FYB	1.77116	LOC100287497*	1.23663
CCDC186	1.75240	MAPK8IP3	1.23174
EEA1	1.70943	JUND	1.21595
CEP290	1.70587	PRKCB	1.21453
TFPI2	1.69031	PNMA6A	1.20682
SPINK2	1.68987	TRA2A	1.20579
WASF2	1.68907	MIR142	1.20373
SMC3	1.67161	CCDC42B	1.20085
CLIP1	1.67049	RFFL	1.19198
TPR	1.64159	MAST2	1.19130
SSPN	1.64121	MAZ	1.17289
SLX4IP	1.63570	LINC00491	1.16714
WASL	1.63299	FAM129C	1.15564
FANCM	1.62707	SRRM2	1.15540
BOD1L1	1.61953	LOC441124	1.15500
FCHO2	1.59444	MTA1	1.15143
BAG4	1.58071	PNPLA2	1.15122
UBXN4	1.58003	WDFY2	1.15053
SMC1A	1.57081	INO80B	1.14429
YTHDC2	1.57063	MAGEA1	1.14412
PCM1	1.57007	SOX6	1.14046
RASEF	1.56196	AP2A1	1.13672

Supplementary Table 1. Top30 up-regulated genes ($\log_2$ fold change, $\log_2$ FC) in ≥ 65-year-old DLBCL patients resistant to R-CHOP therapy in GSE10846 dataset. p-value≤0.001.

*These genes have not been considered for further analysis due to the unavailability of droplet digital PCR probes or because being long non-coding RNAs.

Responder			Non-responder		
Signature	p-value	n. molecules	Signature	p-value	n. molecules
Head and neck neoplasia	2.68e-8	27	Head and neck neoplasia	5.68e-6	22
Hematologic cancer	1.07e-5	16	Expression of RNA	3.00e-3	8
Tumorigenesis of lymphocytes	5.80e-4	11	Transcription of RNA	3.01e-3	7
B cell cancer	9.02e-4	9	Proliferation of B lymphocytes	4.75e-3	3

Supplementary Table 2. Signatures associated with overexpressed genes shown in Supplementary Table 1.

	**Responder**	**Non-responder**		
GENE	**copies/µl (*GENE/GAPDH*)**		**FC (Responder/Non-responder)**	**p-value**
MYO6	0.07905	0.00983	8.04259	0.00659
EBF1	0.46081	0.19387	2.37685	0.02191
CCDC186	0.03814	0.02332	1.63571	0.12342
THRAP3	1.02953	0.77228	1.33310	0.22693
FYB	1.64093	1.49881	1.09482	0.36798
CCDC18	0.28333	0.27741	1.02136	0.48612
EDNRA	0.32588	0.30521	1.06772	0.46501
MIS18BP1	0.19527	0.19673	0.99255	0.49382
TOP1	0.00392	0.01079	0.36323	0.14619

Supplementary Table 3. Differentially expressed genes in a cohort of over 65-year-old patients resistant to R-CHOP.

	**Responder**	**Non-responder**		
GENE	**copies/µl (*GENE/GAPDH*)**		**FC (Non-responder/Responder)**	**p-value**
CALR	0.10201	0.13929	1.36543	0.13600
ATAD3A	0.04650	0.04039	0.86861	0.37595
GALT	0.07182	0.05945	0.82774	0.41806
CWF19L1	0.02063	0.01842	0.89292	0.44417
MAPK18IP3	0.02672	0.02381	0.89109	0.35010
SLC38A5	0.28326	0.10637	0.37553	0.08083
MAGEA9	1.10777	0.01325	0.01196	0.01623

Supplementary Table 4. Differentially expressed genes in a cohort of over 65-year-old patients resistant to R-CHOP

References

1. Tang Z, Li C, Kang B, Gao G, Li C, Zhang Z. GEPIA: a web server for cancer and normal gene expression profiling and interactive analyses. Nucleic Acids Res. 2017;45(W1):W98-W102.
