# Supplementary figures and images for: EBF1, MYO6 and CALR expression levels predict therapeutic response in diffuse large B-cell lymphomas
Source: Front Immunol. 2023 Nov 14;14:1266265. doi: 10.3389/fimmu.2023.1266265 (PMC10682075; doi:10.3389/fimmu.2023.1266265)

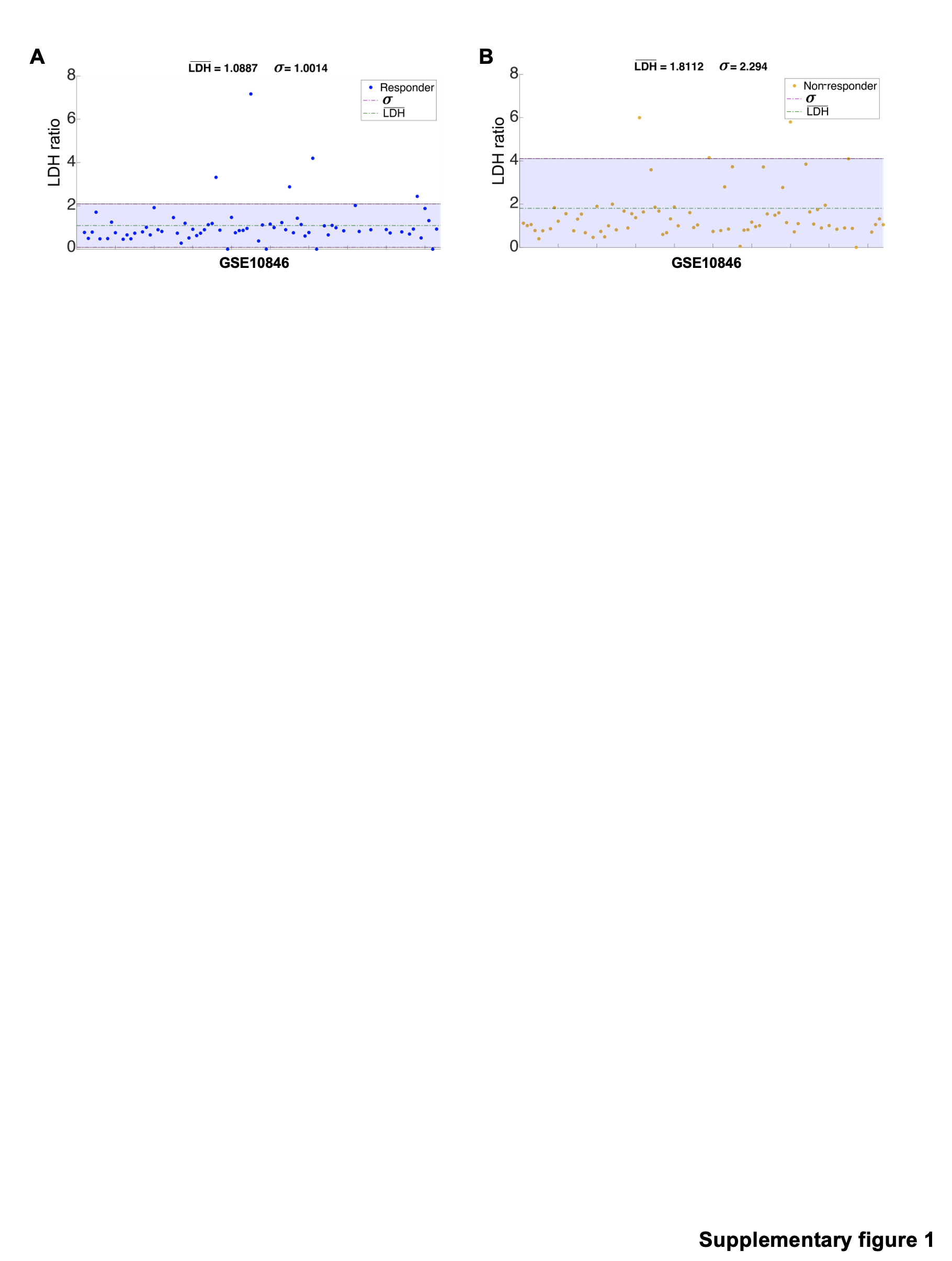

Supplement: Supplementary file 2 [file Image_1.tiff]

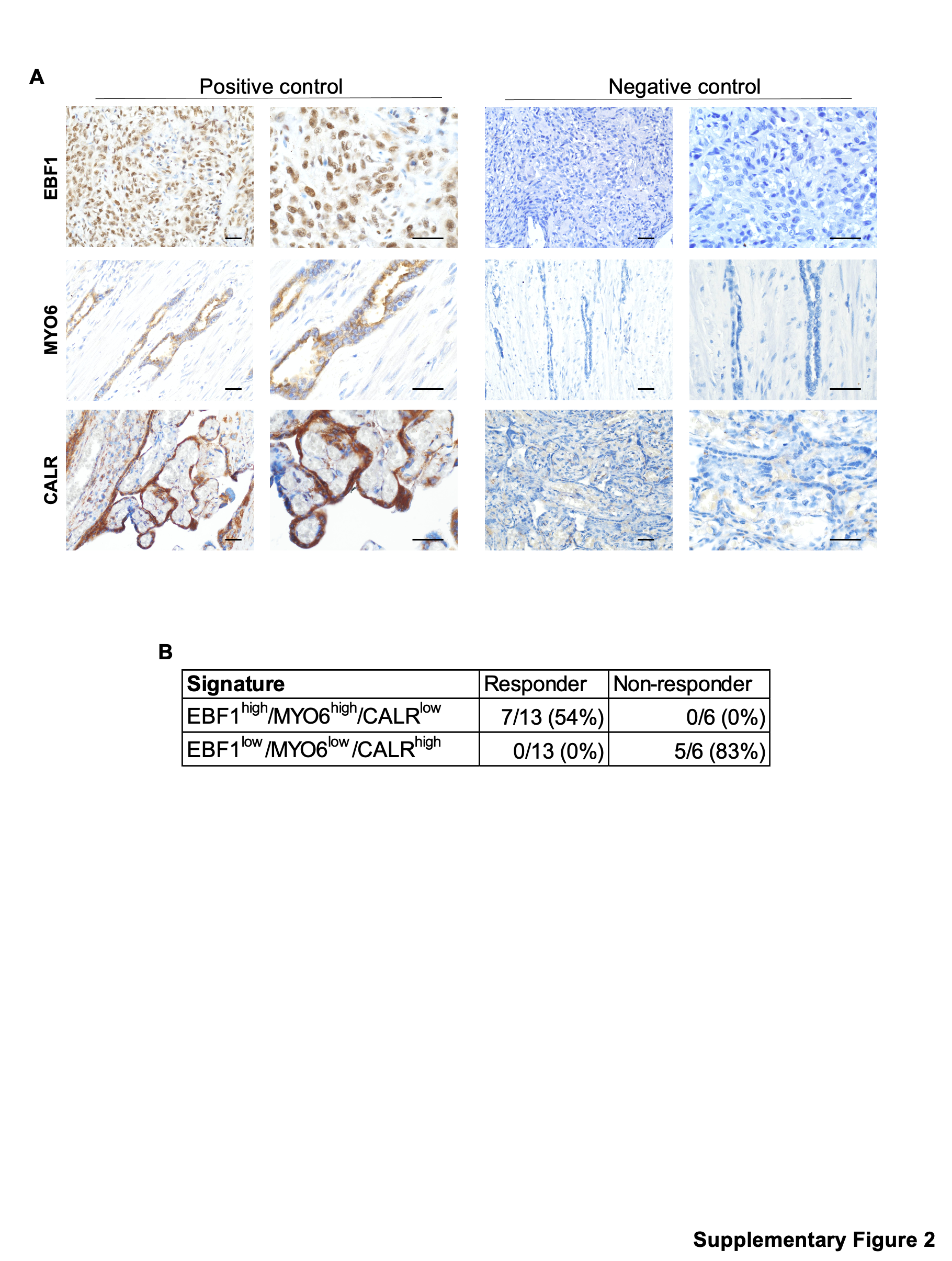

Supplement: Supplementary file 3 [file Image_2.tiff]

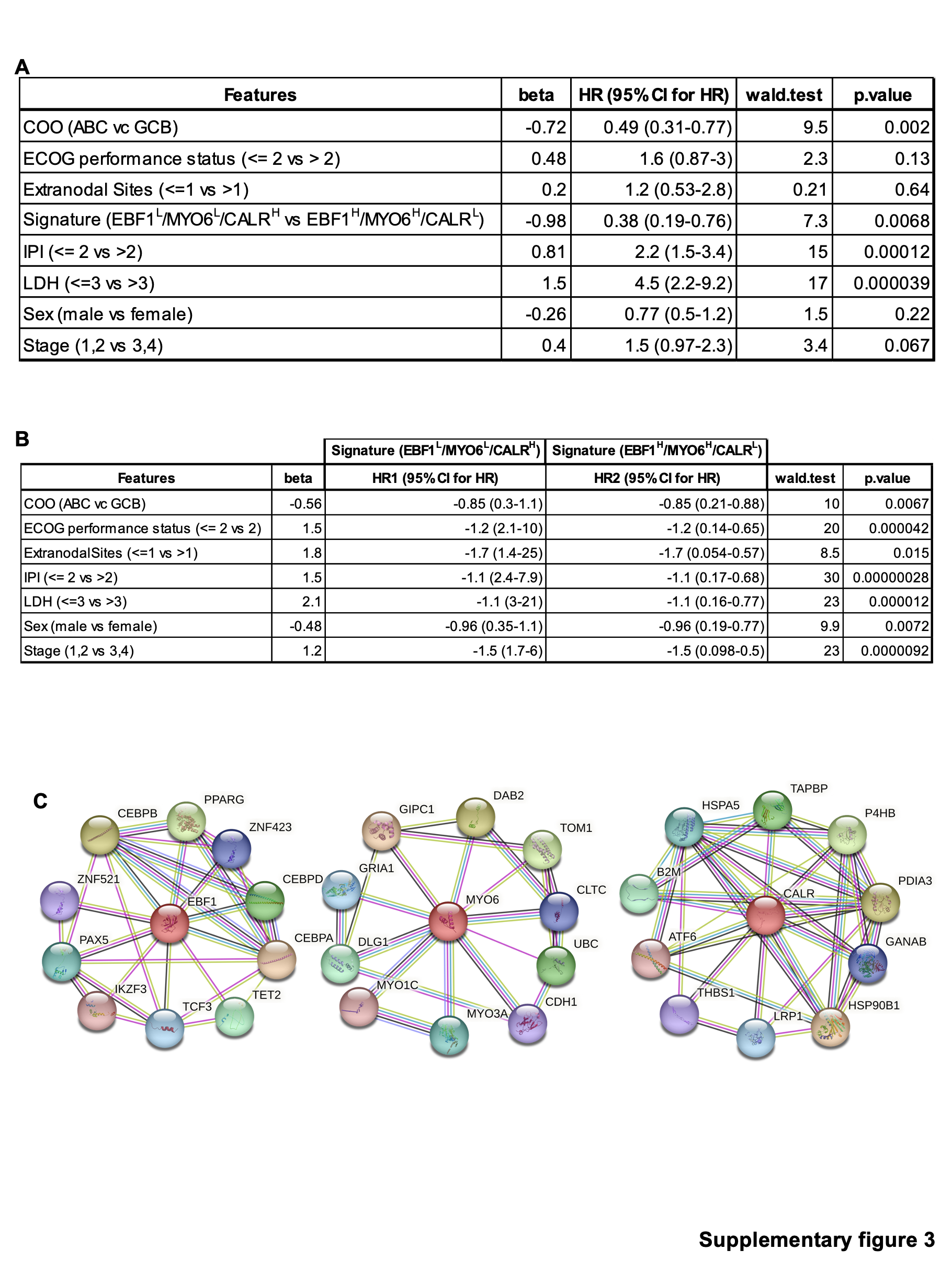

Supplement: Supplementary file 4 [file Image_3.tiff]

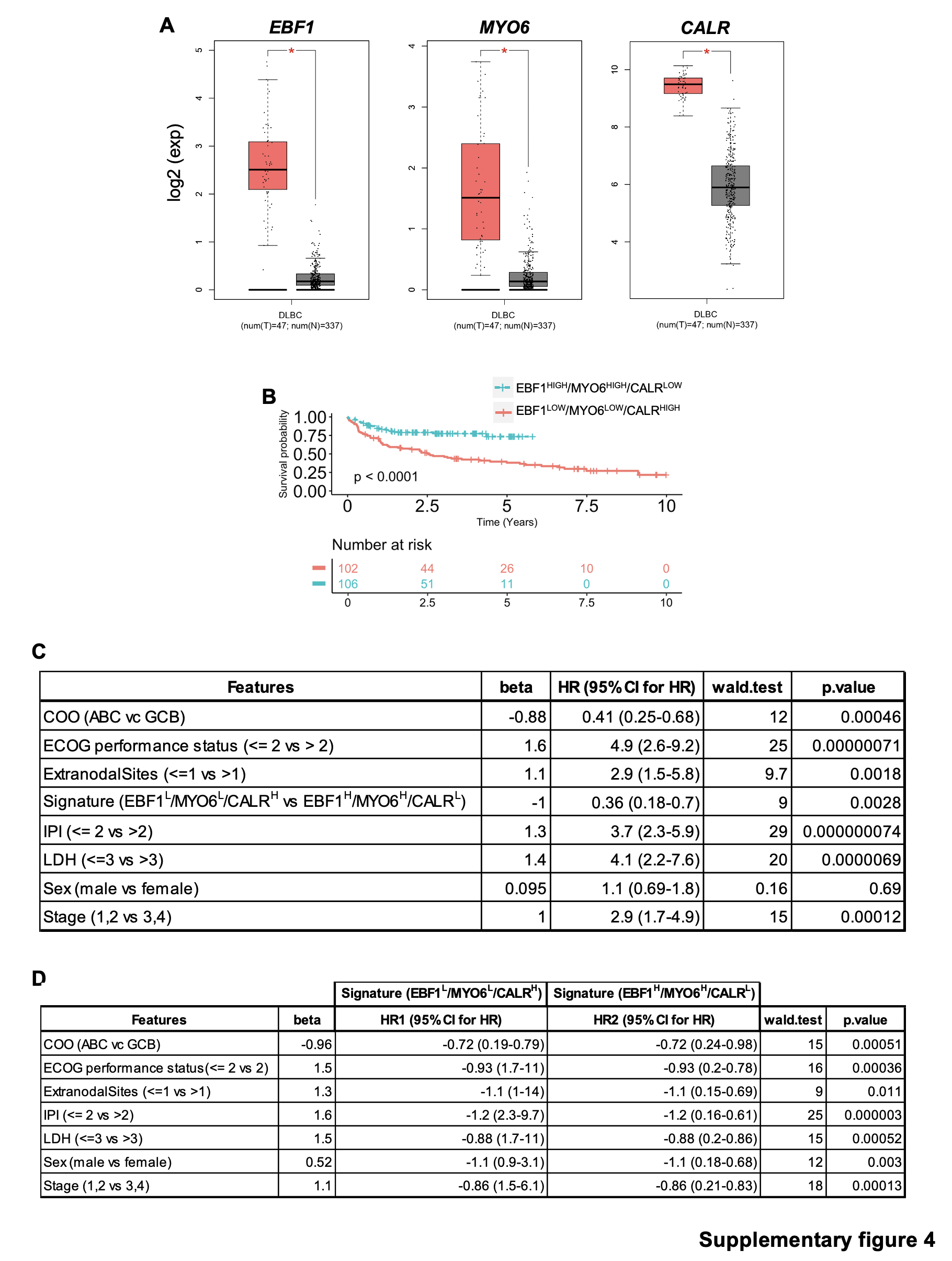

Supplement: Supplementary file 5 [file Image_4.tiff]
